# An *LW-Opsin* Mutation Changes the Gene Expression of the Phototransduction Pathway: A *Cryptochrome1* Mutation Enhances the Phototaxis of Male *Plutella xylostella* (Lepidoptera: Plutellidae)

**DOI:** 10.3390/insects14010072

**Published:** 2023-01-12

**Authors:** Shao-Ping Chen, Xiao-Lu Lin, Rong-Zhou Qiu, Mei-Xiang Chi, Guang Yang

**Affiliations:** 1 State Key Laboratory of Ecological Pest Control for Fujian and Taiwan Crops, Institute of Applied Ecology, Fujian Agriculture and Forestry University, Fuzhou 350002, China; 2 Joint International Research Laboratory of Ecological Pest Control, Ministry of Education, Fuzhou 350002, China; 3 Ministerial and Provincial Joint Innovation Centre for Safety Production of Cross-Strait Crops, Fujian Agriculture and Forestry University, Fuzhou 350002, China; 4 Key Laboratory of Integrated Pest Management for Fujian-Taiwan Crops, Ministry of Agriculture, Fuzhou 350002, China; 5 Key Laboratory of Green Pest Control, Fujian Province University, Fuzhou 350002, China; 6 Fujian Key Laboratory for Monitoring and Integrated Management of Crop Pests, Institute of Plant Protection, Fujian Academy of Agricultural Sciences, Fuzhou 350013, China

**Keywords:** *Plutella xylostella*, *LW-opsin*, phototransduction, *cryptochrome1*, phototactic behavior

## Abstract

**Simple Summary:**

The Diamondback moth (DBM, *Plutella xylostella*) is a worldwide destructive pest with a typical phototaxis, mainly damaging cruciferous vegetables. Our previous study revealed that an *LW-opsin* mutation causes a defective phototaxis in *P*. *xylostella*, but the mechanism behind this phenomenon remains unknown. In this study, the head transcriptomes of the male Geneva 88 strain of *P*. *xylostella* (G88) and an *LW-opsin* mutant were compared to reveal the expression changes of other genes in the phototransduction pathway caused by the mutation of *LW-opsin*. The results showed that the *LW-opsin* mutation caused expression changes of the genes in the phototransduction pathway, such as *neither inactivation nor afterpotential C* (*ninaC*), *retinal degeneration C* (*rdgC*), *arrestin1* (*arr1*), *cryptochrome1* (*cry1*), *transient receptor potential* (*trp*), *transient receptor potential like* (*trpl*) and *inactivation nor afterpotential D* (*inaD*). Considering CRY1 acts as a UV/blue-light photoreceptor, the influence of a *cry1* mutation on the phototaxis of *P. xylostella* was examined, and the results showed that the male *cry1* mutant possessed higher phototactic rates to UV and blue lights than the male G88. Our results provide a foundation for further exploration of the phototransduction pathway of *P. xylostella*.

**Abstract:**

*Plutella xylostella* is a typical phototactic pest. *LW-opsin* contributes to the phototaxis of *P. xylostella*, but the expression changes of other genes in the phototransduction pathway caused by the mutation of *LW-opsin* remain unknown. In the study, the head transcriptomes of male G88 and *LW-opsin* mutants were compared. A GO-function annotation showed that DEGs mainly belonged to the categories of molecular functions, biological processes, and cell composition. Additionally, a KEGG-pathway analysis suggested that DEGs were significantly enriched in some classical pathways, such as the phototransduction-fly and vitamin digestion and absorption pathways. The mRNA expressions of genes in the phototransduction-fly pathway, such as *Gq*, *ninaC,* and *rdgC* were significantly up-regulated, and *trp*, *trpl*, *inaD*, *cry1*, *ninaA* and *arr1* were significantly down-regulated. The expression trends of nine DEGs in the phototransduction pathway confirmed by a RT-qPCR were consistent with transcriptomic data. In addition, the influence of a *cry1* mutation on the phototaxis of *P. xylostella* was examined, and the results showed that the male *cry1* mutant exhibited higher phototactic rates to UV and blue lights than the male G88. Our results indicated that the *LW-opsin* mutation changed the expression of genes in the phototransduction pathway, and the mutation of *cry1* enhanced the phototaxis of a *P. xylostella* male, providing a basis for further investigation on the phototransduction pathway in *P. xylostella*.

## 1. Introduction

The visual system is the primary sensory system for insects to perceive light signals. This perception system is realized through the phototransduction pathway, which occurs in specialized neurons known as photoreceptor cells [1]. The *Drosophila* phototransduction pathway is a paradigm for studying signal transduction. Firstly, the photoisomerization of rhodopsin to meta-rhodopsin activates a heterotrimer of the G protein (Gq) via a GDP–GTP exchange, releasing active Gαq; then, phospholipase C (PLC) is activated, which hydrolyzes phosphatidyl-inositol 4,5 bisphosphate (PIP_2_) to generate inositol 1,4,5 trisphosphate (InsP_3_), diacylglycerol (DAG) and a proton. This leads to the opening of the transient receptor potential (TRP) and transient receptor potential-like (TRPL) light-sensitive channels and the Ca^2+^ and Na^+^-permeable channels, causing a depolarization of the photoreceptor cell membrane, thereby completing the conversion from optical stimulus to electrical signals [2,3,4,5,6,7]; however, this remains largely unknown on the phototransduction pathways in other insects.

Currently, studies focusing on the phototransduction pathway in other insects are mainly about the expression patterns of homologous genes associated with the *Drosophila* phototransduction pathway in the insect head or gene-expression changes in insects being stimulated by external light using transcriptomic sequencing technology. For instance, about 20 genes, including *LW-opsin*, *arr1* (*arrestin1*), *arr2*, *trp* and *trpl*, have been identified in the head of *Ptomaphagus hirtus* (Tellkampf, 1844) (troglobiont beetle, Coleoptera: Leiodidae) [8]. In *Bicyclus anynana* (Butler, 1879) (small brown butterfly, Lepidoptera: Nymphalidae), eight genes, such as *arr2*, *BRh* (i.e., *BL-opsin*) and *rdgC* (*retinal degeneration C*), adapt to changes in expression with the seasons [9]. The expression levels of genes related to the phototransduction pathway are changed, for example, when *Mythimna separata* (Walker, 1865) (oriental armyworm, Lepidoptera: Noctuidae) is exposed to different light environments [10]. Additionally, the phototransduction pathway of Lepidoptera is roughly similar to that of *Drosophila* [11]. The gene expressions between butterflies and moths are different, however, which may be due to different habitats, such as that seen in the expression level of *trp* in butterflies which is about 50 times higher than that in moths [11].

Opsins belong to the subfamily of G-protein-coupled receptors (GPCR), the leading membrane protein in photoreceptor cells [6,12]. Opsins covalently bind to small molecular chromophores to form light-sensitive photopigments—rhodopsins—which initiate the phototransduction cascade [2,4]. Opsins contribute to phototactic behaviors in insects, such as *Drosophila* [13], *Nephotettix cincticeps* (Uhler, 1896) (rice green leafhopper, Hemiptera: Cicadellidae) [14], *Spodoptera exigua* (Hübner, 1808) (beet armyworm, Lepidoptera: Noctuidae) [15], *Diaphorina citri* (Kuwayama, 1908) (Asian citrus psyllid, Hemiptera: Liviidae) [16], and *Plutella xylostella* (Linnaeus, 1758) (diamondback moth, Lepidoptera: Plutellidae) [17]. These studies, however, only focus on the function of opsin, and research is lacking on changes in the expression levels of other genes in the phototransduction pathway caused by an opsin mutation.

*Plutella xylostella* is a typical phototactic pest, which is one of the most destructive pests of crucifers worldwide. The mutation of *LW-opsin* results in the defective phototaxis of *P*. *xylostella* [17]. The changes in the expression levels of other genes in the phototransduction pathway caused by the opsin mutation remain unknown in *P*. *xylostella*. In our study, the head transcriptomes of the G88 and *LW-opsin* mutation strains were compared to explore further the influence of an *LW-opsin* mutation on the expression of other genes in the phototransduction pathway. Moreover, the function in the phototaxis of a candidate gene with the expression level influenced by an *LW-opsin* mutation was also verified. Our study contributes to further exploration of the phototransduction pathway in *P. xylostella* and provides theoretical guidance for the behavioral regulation of *P. xylostella*.

## 2. Materials and Methods

### 2.1. Insect Strains and Rearing

Three *P*. *xylostella* strains were used in the study. Geneva 88 (G88) was employed as a wild type, and the other two mutant strains, namely, LW-13 (a 13-bp deletion in *LW-opsin*) [17] and Cry1-KO (a 2-bp deletion in *cryptochrome1*) [18] were developed in a G88 background by using the CRISPR/Cas9 technology. The larvae were reared on an artificial diet (Frontier Scientific Services, Newark, DE, USA), and the adults were fed with a 10% honey solution. The insects were maintained under 26 ± 1 °C, 60% ± 10% relative humidity, and a 14: 10 h (light: dark) photoperiod.

### 2.2. RNA Extraction and Transcriptome-Sequencing

About 30 heads of 2- to 3-day-old male adults of G88 and LW-13 were collected separately for transcriptome-sequencing, which had been in a totally-dark environment for 2–3 h, and then under a green light (520 ± 5 nm, 2.5 lux) for 10 min. The total RNA was extracted by using the Eastep^®^ Super Total 105 RNA Extraction Kit (Promega, Madison, WI, USA), following the manufacturer’s protocol. The RNA was purified via a phenol/chloroform/isoamyl alcohol (25:24:1) extraction because of the high pigment content in the head. The integrity of the purified products was further examined by agarose gel electrophoresis, and the concentration and purity were examined by a NanoDrop2000 (Thermo Fisher, Waltham, MA, USA). The RNA integrity number (RIN) was further examined using a RNA 6000 Nano Lab Chip Kit and Bioanalyzer 2100 (Agilent Technologies, Santa Clara, CA, USA). The samples (RIN ≥ 8.0) were entrusted to the Shanghai Meiji Biological Company for a library construction and sequencing (Illumina Novaseq 6000, San Diego, CA, USA). All the experiments were repeated three times.

### 2.3. Bioinformatic Analyses

Adapter trimming and low-quality filtering of the raw reads were performed by using the SeqPrep (https://github.com/jstjohn/SeqPrep, accessed on 21 November 2022) and Sickle (https://github.com/najoshi/sickle, accessed on 21 November 2022). Then, the clean reads were aligned to the *P*. *xylostella* genome (http://59.79.254.1/DBM/index.php, accessed on 21 November 2022) by using the Bowtie2 (Version 2.4.1) and HISAT2 (http://ccb.jhu.edu/software/hisat2/index.shtml, accessed on 21 November 2022) for sequence analysis. The mapped reads were assembled and spliced using Cufflinks (http://coletrapnelllab.github.io/cufflinks/, accessed on 21 November 2022) or StringTie (http://ccb.jhu.edu/software/stringtie/, accessed on 21 November 2022) based on the *P*. *xylostella* genome. New transcripts were obtained by comparing them with known transcripts using Gffcompare. To avoid omissions caused by annotation errors, the protein sequences of related genes in the photoreceptor pathway of *Drosophila* that have been published on NCBI were collected and used as the query for the BLASTp to identify the potential genes involved in the phototransduction pathway of *P*. *xylostella*.

The differentially-expressed genes (DEGs) between G88 and LW-13 were identified based on transcripts per million reads (TPM) by using RSEM (version 1.3.3) and DESeq2 (version 1.24.0). A Pearson correlation analysis was performed to compare the correlation between the samples. If the *Q* value was less than 0.05 in the multi-group comparison, and the fold of the up- or down-regulation difference was more than one fold, a significant difference in the expression was considered. The enrichment analysis of the gene ontology (GO) and Kyoto encyclopedia of genes and genomes (KEGG) pathway was performed by using the Omicshare platform (https://www.omicshare.com/, accessed on 21 November 2022). The GO and KEGG pathway were considered as a significant enrichment when the *Q* value was less than 0.05.

### 2.4. Validation of Differentially-Expressed Genes by RT-qPCR

The screened DEGs belonging to the phototransduction pathway were selected for a RT-qPCR (Quantitative RT-PCR) to validate the results of the transcriptome sequencing. The amplification of the RT-qPCR was performed by using the GoTaq^®^ qPCR Master Mix (Promega) with an initial step at 95 °C for 10 min followed by 40 cycles at 95 °C for 15 s and 60 °C for 30 s, one cycle at 95 °C for 15 s, and 60 °C for 60 s, and then at 95 °C for 15 s. The gene *RPL32* of *P. xylostella* was used as the reference gene [19]. The gene primers are listed in Table S1. The comparative Ct method (2^−ΔCt^) was applied to calculate the transcript level [20].

### 2.5. Phototactic Behavioral Assays

A *cry1*-knockout strain (Cry1-KO) was used to verify whether a *cry1* mutation affects the *P*. *xylostella* male phototactic behavior. A UV light (380 nm) and blue light (470 ± 10 nm) were used in the phototactic-behavior assays for CRY1 acting as a UV/blue-light photoreceptor [21,22]. The light intensity was 2.5 lux and the phototactic rate (%) = (the number of months in the illuminated room/total number of moths for the experiment) × 100. The details of the phototactic-behavior assays have been described previously [17]. All the experiments were repeated four times.

### 2.6. Statistical Analysis

All the statistical analyses were conducted using the SPSS software (version 22.0, IBM, Armonk, NY, USA). An independent *t*-test was applied to determine the significant differences in the gene expression between the RT-qPCR and transcriptome sequencing. The Mann–Whitney *U* test was employed to test the significant differences in the phototactic rate between G88 and Cry1-KO.

## 3. Results

### 3.1. Transcriptome-Sequencing Data Analysis

**Figure 1:**
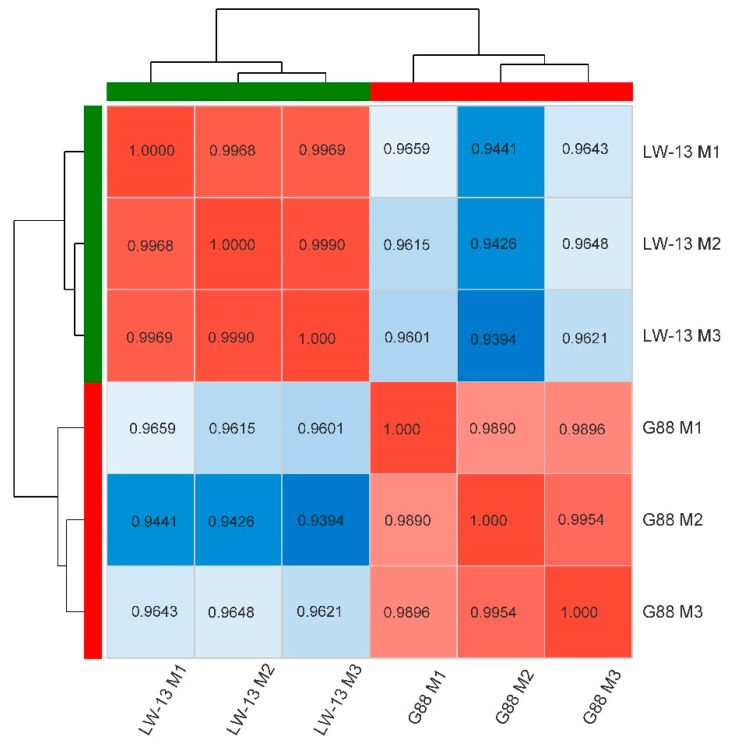
Correlation coefficient heatmap of gene expressions between different samples of *P. xylostella*. The green bar outside the heatmap represents the LW-13 group; the red bar outside the heatmap represents the G88 group; the values in square frames with red and blue color represent the Pearson correlation coefficients of LW-13 and G88, respectively; the darker color indicates the bigger value.

Approximately 39.23 Gb of clean data were obtained, and the clean data of each sample was more than 6.13 Gb. In total, 24,186 genes and 37,220 transcripts were detected. The annotated genes and transcripts were 14,590 and 12,938, respectively. About 55.05% of high-quality clean data remained for the assembly and further analysis (Table 1). The low-total mapped ratios in Table 1 might be because the genome of the Fuzhou strain (a heterozygous genome) [23] was used as the reference for analyzing the transcriptomic data from G88 and its mutant. The *Q*_30_ of each sample was more than 94.61% (Table 1), indicating that the transcriptome-sequencing data was qualified. The correlation of the samples between the biological replicate samples was more significant than that between the G88 and LW-13, and the Pearson correlation coefficients between the biological replicate samples in each group were all greater than 98.90% (Figure 1), indicating that the transcriptome-sequencing data could be used for the subsequent analysis.

### 3.2. Identification of DEGs

**Figure 2:**
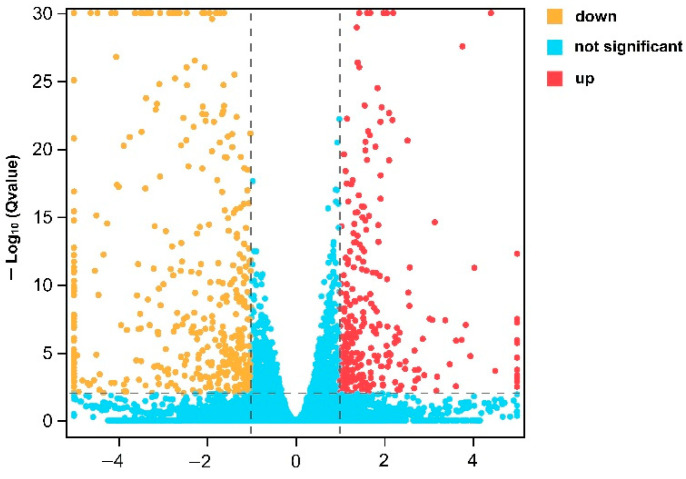
Volcanic map of DEGs in LW-13 compared with G88.

The head transcriptomes of male G88 and LW-13 were compared, and a total of 1890 (7.81%) DEGs were found. Compared with the G88, 898 genes in the LW-13 were significantly up-regulated, and 992 genes were significantly down-regulated (Figure 2), indicating significant differences in the gene expression between these two *P. xylostella* strains.

### 3.3. Gene Ontology of DEGs

The GO cluster diagram of the DEGs showed that the DEGs were mainly enriched in 16 biological processes, such as the metabolic process, cellular process, and localization; in 14 cellular components, such as the cell part, membrane part, and membrane; and in 7 molecular functions, such as in binding, catalytic activity, and transporter activity (Figure S1).

**Figure 3:**
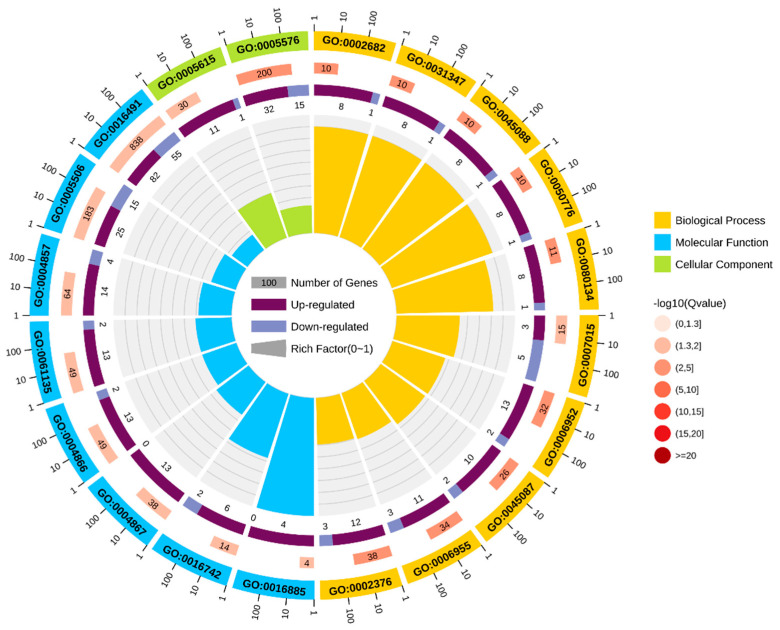
Top 20 GO term enrichment of DEGs in LW-13 compared with G88. From the outside to the inside, the first circle represents the enriched GO classification where the different colors represent different classifications, and the numbers outside the circle represent the coordinate ruler of the number of genes. The second circle represents the number of genes corresponding to the classification and the *Q* value, where the longer the bar indicates a higher number of genes, and the darker red color indicates a smaller *Q* value. The third circle represents the ratio of up- and down-regulated genes, where dark purple represents the up-regulated genes, light purple represents the down-regulated genes, and the numbers of the corresponding bottom represent the up-regulated genes number or down-regulated genes number. The fourth circle represents the rich factor value of each GO term, and each cell represents 0.1.

A top 20 GO term enrichment analysis showed that the DEGs mainly belonged to: the categories of catalytic activity in molecular functions (*Q* < 0.05, Figure 3), such as hydroxymethyl-, formyl- and related transferase activity (GO: 0016742, *Q* = 0.037), oxidoreductase activity (GO: 0016491, *Q* = 0.037), and enzyme inhibitor activity (GO: 0004857, *Q* = 0.045); the categories of metabolic and cellular processes in biological processes (*Q* < 0.05, Figure 3), such as regulation of the immune system process (GO: 0002682, *Q* < 0.001), regulation of the defense response (GO: 0031347, *Q* < 0.001), and actin filament organization (GO: 0007015, *Q* = 0.018); and the categories of cell components and membrane components in cell composition (*Q* < 0.05, Figure 3), including the extracellular region (GO: 0005576, *Q* < 0.001) and extracellular space (GO: 0005615, *Q* = 0.010).

### 3.4. KEGG Pathway of DEGs

**Figure 4:**
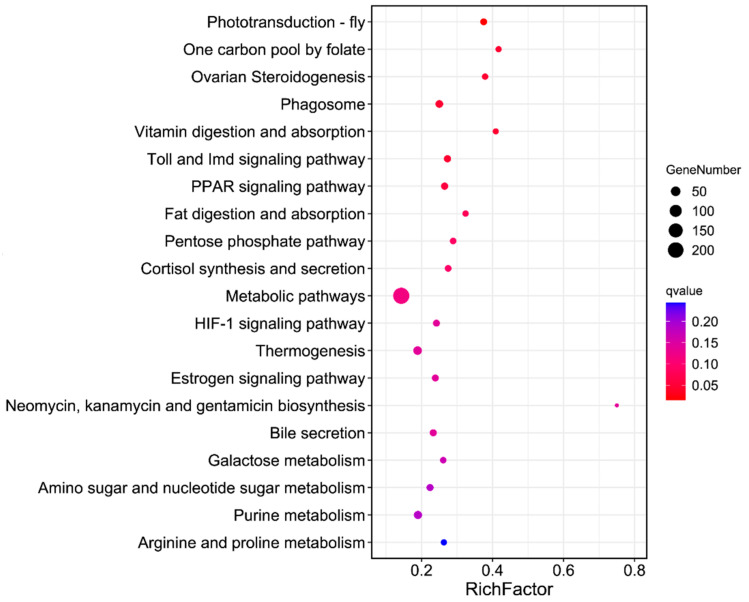
Top 20 of the KEGG enrichment of DEGs in LW-13 compared with G88.

The KEGG pathways analysis suggested that some classical pathways, such as the phototransduction-fly (KO04745, *Q* = 0.01), one carbon pool by folate (KO00670, *Q* = 0.04), ovarian steroidogenesis (KO04913, *Q* = 0.04), phagosome (KO04145, *Q* = 0.04), vitamin digestion and absorption (KO04977, *Q* = 0.04), and toll and Imd signaling pathway (KO04624, *Q* = 0.04), were significantly enriched (Figure 4).

### 3.5. Influences of LW-Opsin Mutation on the Expression of Genes in the Phototransduction Pathway

**Figure 5:**
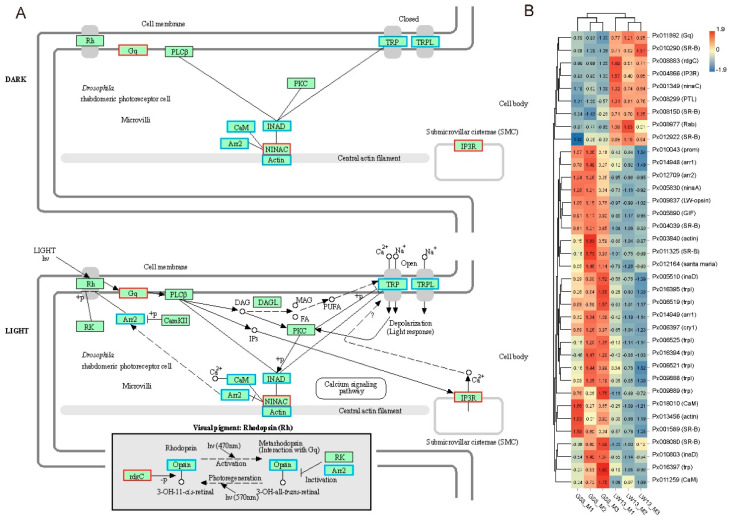
DEGs in LW-13 compared with G88 based on the phototransduction-fly pathway and their heatmap. (**A**) DEGs in LW-13 compared with G88 strains based on the phototransduction-fly pathway. Red and blue frames represent up-regulated and down-regulated DEGs, respectively. (**B**) Heatmap of related DEGs. The value in the box represents the value of the gene’s TPM normalized by the *Z*-score.

Further analysis of the phototransduction-fly pathway revealed that the mRNA expression of the key elements of *Gq*, *IP_3_R* (*InsP_3_ receptor*), *ninaC* (*neither inactivation nor afterpotential C*) and *rdgC* were significantly up-regulated, that the mRNA expression of the key elements of *arr2*, *trp*, *trpl*, *CaM* (*calmodulin*), *actin* and *inaD* (*inactivation nor afterpotential D*) were significantly down-regulated, and that the mRNA expression of the key elements of *PLC*, *PKC* (*protein kinase C*), *CaMKII* (*CaM kinase II*), and *DAGL* (*diacylglycerol lipase*) were not affected (Figure 5A,B). Moreover, other genes that participated in the phototransduction-fly pathway, such as *cry1*, *ninaA* and *arr1*, were also significantly down-regulated (Figure 5B).

### 3.6. RT-qPCR Validation of DEGs in the Phototransduction Pathway

**Figure 6:**
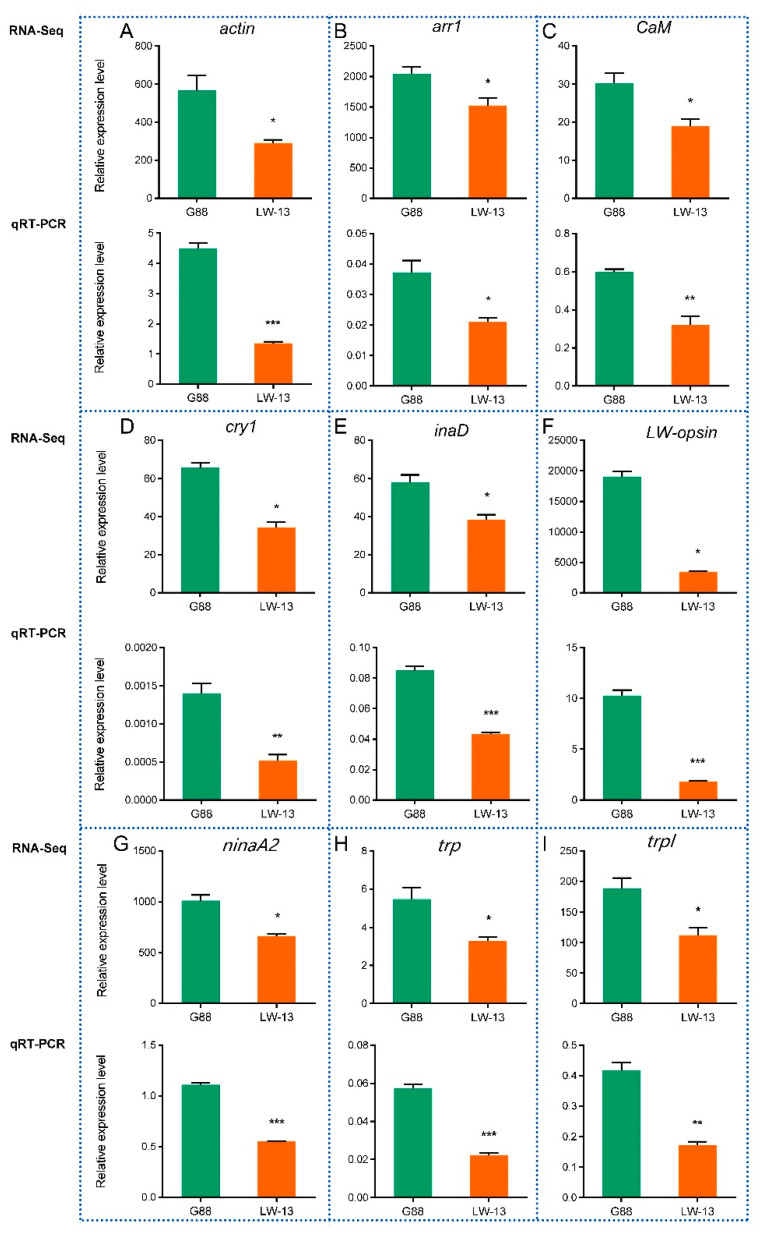
Verification of the expressions of 9 DEGs in LW-3 compared with G88 by RT-qPCR. (**A**) *actin* (gene ID, Px003840); (**B**) *arr1* (gene ID, Px014948); (**C**) *CaM* (gene ID, Px018010); (**D**) *cry1* (gene ID, Px006397); (**E**) *inaD* (gene ID, Px005510); (**F**) *LW-opsin* (gene ID, Px009837; the *LW-opsin* expression in LW-13 is mutant *LW-opsin*); (**G**) *ninaA* (gene ID, Px005830); (**H**) *trp* (gene ID, Px009889); (**I**) *trpl* (gene ID, Px009888). “*” indicates *P* < 0.05; “**” indicates 0.01 < *P* < 0.05; “***” indicates *P* < 0.01.

The gene expression levels of the nine genes of *actin*, *arr1*, *CaM*, *cry1*, *inaD*, *LW-opsin*, *ninaA*, *trp* and *trpl* in the phototransduction pathway by the RT-qPCR were consistent with the transcriptome-sequencing results in G88 and LW-13 (Figure 6; *r* = 0.9136, *p* = 0.0006). It was worth noting that the *LW-opsin* mutation caused a remarkably lower mRNA expression of mutant *LW-opsin*, indicating the reliability of our transcriptomic data.

### 3.7. The Effects of cry1 Mutation on Phototactic Behavior of P. xylostella

**Figure 7:**
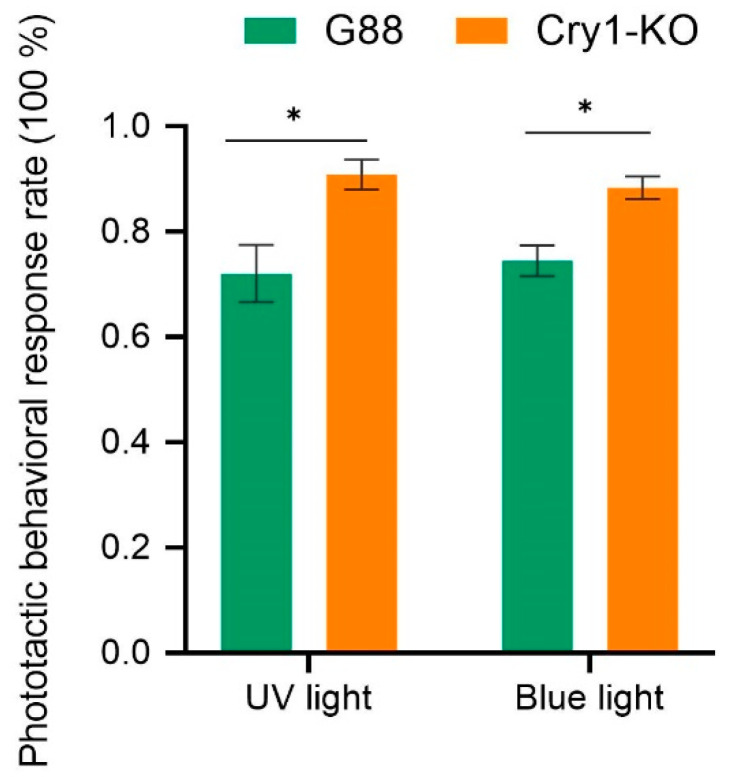
The influence of *cry1* mutation on the phototaxis of *P. xylostella* male. “*” represents *P* < 0.05.

The male *P. xylostella* with a 2-bp deletion in *cry1* (Cry1-KO) showed higher phototactic rates to UV and blue lights than the male G88 (Figure 7).

## 4. Discussion

*LW-opsin* is a gene with an absolute dominance expression compared with other opsins in *P*. *xylostella* [17]. Differentially-expressed genes caused by an *LW-opsin* mutation in *P*. *xylostella* mainly enriched in the GO terms, such as the actin filament organization, extracellular region, and extracellular space, confirming that opsins are required to maintain photoreceptor integrity [24,25,26]. Further work on observing the internal morphological characteristics of photoreceptors using a transmission electron microscope (TEM) will be needed. Although opsins are known as light sensors, some of them (Rh1 and Rh6) also contribute to locomotion in *Drosophila* [27]. In our study, DEGs were also enriched in the locomotion GO term (Figure S1), and this function had been preliminarily verified in our previous work [17], although this result needs to be confirmed by a proprioceptor dissection.

The phototransduction-fly pathway was significantly enriched after an *LW-opsin* mutation, indicating the *P*. *xylostella* phototransduction pathway was similar to that in *Drosophila*. In *P*. *xylostella*, the *LW-opsin* mutation affected the mRNA expression of some key elements, such as *arr2*, *inaD*, *rdgC*, *trp* and *trpl*; but the mRNA expression of some other vital elements, such as *PLC* and *PKC*, were not affected. The results suggest that there are variations to accommodate the visual requirements for *P*. *xylostella* compared with *Drosophila*, which might be due to the different lifestyles and eye morphologies between the two insect species. The functions of these critical elements have been well demonstrated in *Drosophila*. For instance, Gq is an essential effector in the activation of PLC, and the mutant shows a decreased light sensitivity [28]. Arr2 is an inhibitory protein responsible for inactivation of rhodopsin and plays an essential role in terminating the phototransduction response, and a mutation of *arr2* results in retinal degeneration [29]. Additionally, INAD is a scaffold protein containing five PDZ structural domains, which can directly bind PKC, PLC, TRP, TRPL, and NINAC, and a mutation of *inaD* results in terminating the phototransduction response [30,31,32,33]. Finally, TRP and TRPL are cation channels, and a mutation of *trp* and *trpl* results in a decreased Ca^2+^ influx and transient response to light [34,35]. The function of these key elements in *P*. *xylostella* will be further verified by using RNAi or CRISPR/Cas9 technology.

The vitamin digestion and absorption pathway was also significantly enriched, which might have been because vitamin A is a critical substrate in chromophore biogenesis [26,36,37,38,39]. Whether an *LW-opsin* mutation in *P. xylostella* disrupts chromophore production will be further explored by using HPLC-MS (high-performance liquid chromatography-mass spectrometry). Moreover, vitamin A is essential for an immune response [40,41], which might be the reason for the DEGs’ enrichment in GO terms, such as regulation of the immune system process and the KEGG pathway–Toll and Imd signaling pathway.

Higher phototaxis responses to the UV and blue lights were observed in the male *P. xylostella cry1* mutants than in the G88. It has previously been assumed that the ability of an insect to sense and respond to UV light is regulated by UV-opsin [21]; however, UV-opsin does not participate in sensing UV light in *P*. *xylostella* [17]. Insects express other non-opsin photoreceptors, including the UV/blue-light sensitive flavoprotein, CRY [21,22]. The dCRY is a significant modulator of *Drosophila* behavioral responses to UV light, containing both attraction and avoidance behaviors. *Drosophila* shows avoidance behaviors to high-intensity UV light (400 μW/cm^2^), while showing attraction behaviors to low-intensity UV light (10 μW/cm^2^) [42]. Conversely, mutant *Drosophila* lacking CRY showed attraction behaviors instead of avoidance behaviors to high-intensity UV light and more attraction to low-intensity UV light than wild-type control flies [21,42]. Although further studies are needed to verify whether *P. xylostella* shows avoidance behaviors to high-intensity UV light or blue light, our results suggested that CRY1 might be a significant modulator of the *P. xylostella* behavioral responses to low-intensity UV light (380 nm and 2.5 lux ≈ 60 μW/cm^2^) and blue light (470 nm and 2.5 lux ≈ 4 μW/cm^2^).

Interestingly, the phototaxis of female *P. xylostella cry1* mutants to UV and blue lights showed no difference with the G88 (Figure S2). A gender difference in the phototaxis behavior was also observed in the *P. xylostella LW-opsin* mutant [17]. Moreover, the *P. xylostella* males exhibited a better locomotor circadian rhythm (induced by light) than the females [43]. These results suggest that the phototransduction mechanism might be diverse between *P. xylostella* male and females.

In *Drosophila*, the dCRY C-terminal interacts with NINAC through INAD in a light-dependent manner [44]. In our study, the *LW-opsin* mutation resulted in lower expressions of *cry1* and *inaD*, and a higher expression of *ninaC*. These results suggested that the behavioral response to the UV light mediated by CRY1 might be similar between *Drosophila* and *P. xylostella* males; however, the expression of *inaD* and *ninaC* in Cry1-KO will need further verification.

## 5. Conclusions

In conclusion, an *LW-opsin* mutation causes gene expressions in the phototransduction pathway, such as *arr1*, *cry1*, *trp*, *trpl* and *inaD*, and *cry1* plays an essential role in enhancing the phototaxis of *P. xylostella* males. Our results provide a preliminary system-level understanding of the influences of the *LW-opsin* mutation on the expression of genes in *P*. *xylostella* phototransduction and they provide a foundation for further exploration of the phototactic mechanisms in *P. xylostella*.

## Figures and Tables

**Table 1:** Summary of transcriptome-sequencing results generated from G88 and LW-13.

Sample	*Q*_30_ (%)	Total Mapped	Multiple Mapped	Uniquely Mapped
G88 M1	94.61	23,206,466 (55.63%)	1,350,166 (3.24%)	21,856,300 (52.39%)
G88 M2	94.67	25,289,426 (55.93%)	1,448,410 (3.20%)	23,841,016 (52.72%)
G88 M3	94.86	25,923,219 (56.01%)	1,521,146 (3.29%)	24,402,073 (52.72%)
LW-13 M1	94.67	22,971,282 (54.15%)	1,367,482 (3.22%)	21,603,800 (50.92%)
LW-13 M2	94.81	23,454,846 (54.49%)	1,393,748 (3.24%)	22,061,098 (51.25%)
LW-13 M3	94.76	26,024,544 (54.10%)	1,496,406 (3.11%)	24,528,138 (50.99%)

## Data Availability

The data presented in this study are available on request from the corresponding author.

## Supplementary Materials

The following supporting information can be downloaded at: https://www.mdpi.com/article/10.3390/insects14010072/s1, Figure S1: GO cluster diagram of DEGs; Figure S2: The influence of *cry1* mutation on the phototaxis of *P. xylostella* female; Table S1: RT-qPCR primers.

## References

[1] Fain G.L., Hardie R., Laughlin S.B. (2010). Phototransduction and the evolution of photoreceptors. Curr. Biol..

[2] Hardie R.C., Juusola M. (2015). Phototransduction in *Drosophila*. Curr. Opin. Neurobiol..

[3] Katz B., Minke B. (2018). The *Drosophila* light-activated TRP and TRPL channels—Targets of the phosphoinositide signaling cascade. Prog. Retin. Eye Res..

[4] Landry C.R., Castillo-Davis C.I., Ogura A., Liu J.S., Hartl D.L. (2007). Systems-level analysis and evolution of the phototransduction network in *Drosophila*. Proc. Natl. Acad. Sci. USA.

[5] Montell C. (2012). *Drosophila* visual transduction. Trends Neurosci..

[6] Paulsen R., Schwemer J. (1983). Biogenesis of blowfly photoreceptor membranes is regulated by 11-*cis*-retinal. Eur. J. Biochem..

[7] Yau K.W., Hardie R.C. (2009). Phototransduction motifs and variations. Cell.

[8] Friedrich M., Chen R., Daines B., Bao R., Caravas J., Rai P.K., Zagmajster M., Peck S.B. (2011). Phototransduction and clock gene expression in the troglobiont beetle *Ptomaphagus hirtus* of Mammoth cave. J. Exp. Biol..

[9] Macias-Munoz A., Smith G., Monteiro A., Briscoe A.D. (2016). Transcriptome-wide differential gene expression in *Bicyclus anynana* butterflies: Female vision-related genes are more plastic. Mol. Biol. Evol..

[10] Duan Y., Gong Z.J., Wu R.H., Miao J., Jiang Y.L., Li T., Wu X.B., Wu Y.Q. (2017). Transcriptome analysis of molecular mechanisms responsible for light-stress response in *Mythimna separata* (Walker). Sci. Rep..

[11] Macias-Muñoz A., Rangel Olguin A.G., Briscoe A.D., Li W.H. (2019). Evolution of phototransduction genes in lepidoptera. Genome Biol. Evol..

[12] Feuda R., Marletaz F., Bentley M.A., Holland P.W.H. (2016). Conservation, duplication, and divergence of five opsin genes in insect evolution. Genome Biol. Evol..

[13] Yamaguchi S., Desplan C., Heisenberg M. (2010). Contribution of photoreceptor subtypes to spectral wavelength preference in *Drosophila*. Proc. Natl. Acad. Sci. USA.

[14] Wakakuwa M., Stewart F., Matsumoto Y., Matsunaga S., Arikawa K. (2014). Physiological basis of phototaxis to near-infrared light in *Nephotettix cincticeps*. J. Comp. Physiol. A.

[15] Liu Y.J., Yan S., Shen Z.J., Li Z., Zhang X.F., Liu X.M., Zhang Q.W., Liu X.X. (2018). The expression of three opsin genes and phototactic behavior of *Spodoptera exigua* (Lepidoptera: Noctuidae): Evidence for visual function of opsin in phototaxis. Insect Biochem. Mol. Biol..

[16] Li C., Tian F., Lin T., Wang Z., Liu J., Zeng X. (2020). The expression and function of opsin genes related to the phototactic behavior of Asian citrus psyllid. Pest Manag. Sci..

[17] Chen S.P., Liu Z.X., Chen Y.T., Wang Y., Chen J.Z., Fu S., Ma W.F., Xia S., Liu D., Wu T. (2021). CRISPR/Cas9-mediated knockout of *LW-opsin* reduces the efficiency of phototaxis in the diamondback moth *Plutella xylostella*. Pest Manag. Sci..

[18] Chen S.P., Wang D.F., Ma W.F., Lin X.L., Yang G. (2022). Knockout of *cryptochrome1* disturbs the locomotor circadian rhythm and development of *Plutella xylostella*. Insect Sci..

[19] Ma X.L., He W.Y., Wang P., You M.S. (2019). Cell lines from diamondback moth exhibiting differential susceptibility to baculovirus infection and expressing midgut genes. Insect Sci..

[20] Livak K.J., Schmittgen T.D. (2001). Analysis of relative gene expression data using real-time quantitative PCR and the 2^−ΔΔCT^ method. Methods.

[21] Baik L.S., Fogle K.J., Roberts L., Galschiodt A.M., Chevez J.A., Recinos Y., Nguy V., Holmes T.C. (2017). CRYPTOCHROME mediates behavioral executive choice in response to UV light. Proc. Natl. Acad. Sci. USA.

[22] Bouly J.P., Schleicher E., Dionisio-Sese M., Vandenbussche F., Van Der Straeten D., Bakrim N., Meier S., Batschauer A., Galland P., Bittl R. (2007). Cryptochrome blue light photoreceptors are activated through interconversion of flavin redox states. J. Biol. Chem..

[23] You M., Yue Z., He W., Yang X., Yang G., Xie M., Zhan D., Baxter S.W., Vasseur L., Gurr G.M. (2013). A heterozygous moth genome provides insights into herbivory and detoxification. Nat. Genet..

[24] Colley N.J., Cassill J.A., Baker E.K., Zuker C.S. (1995). Defective intracellular transport is the molecular basis of rhodopsin-dependent dominant retinal degeneration. Proc. Natl. Acad. Sci. USA.

[25] Kumar J.P., Ready D.F. (1995). Rhodopsin plays an essential structural role in *Drosophila* photoreceptor development. Development.

[26] Wang T., Montell C. (2007). Phototransduction and retinal degeneration in *Drosophila*. Pflug. Arch. Eur. J. Physiol..

[27] Zanini D., Giraldo D., Warren B., Katana R., Andrés M., Reddy S., Pauls S., Schwedhelm-Domeyer N., Geurten B.R.H., Göpfert M.C. (2018). Proprioceptive opsin functions in *Drosophila* larval locomotion. Neuron.

[28] Scott K., Becker A., Sun Y., Hardy R., Zuker C. (1995). Gqα protein function in vivo: Genetic dissection of its role in photoreceptor cell physiology. Neuron.

[29] Dolph P.J., Ranganathan R., Colley N.J., Hardy R.W., Socolich M., Zuker C.S. (1993). Arrestin function in inactivation of G protein-coupled receptor rhodopsin in vivo. Science.

[30] Chevesich J., Kreuz A.J., Montell C. (1997). Requirement for the PDZ domain protein, INAD, for localization of the TRP store-operated channel to a signaling complex. Neuron.

[31] Li H.S., Montell C. (2000). TRP and the PDZ protein, INAD, form the core complex required for retention of the signalplex in *Drosophila* photoreceptor cells. J. Cell Biol..

[32] Wes P.D., Xu X.Z.S., Li H.S., Chien F., Doberstein S.K., Montell C. (1999). Termination of phototransduction requires binding of the NINAC myosin III and the PDZ protein INAD. Nat. Neurosci..

[33] Xu X.Z.S., Choudhury A., Li X., Montell C. (1998). Coordination of an array of signaling proteins through homo- and heteromeric interactions between PDZ domains and target proteins. J. Cell Biol..

[34] Hardie R.C., Minke B. (1992). The *trp* gene is essential for a light-activated Ca^2+^ channel in *Drosophila* photoreceptors. Neuron.

[35] Leung H.T., Geng C., Pak W.L. (2000). Phenotypes of *trpl* mutants and interactions between the transient receptor potential (TRP) and TRP-like channels in *Drosophila*. J. Neurosci..

[36] Dewett D., Lam-Kamath K., Poupault C., Khurana H., Rister J. (2021). Mechanisms of vitamin A metabolism and deficiency in the mammalian and fly visual system. Dev. Biol..

[37] Dewett D., Labaf M., Lam-Kamath K., Zarringhalam K., Rister J. (2021). Vitamin A deficiency affects gene expression in the *Drosophila melanogaster* head. G3 Genes Genomes Genet..

[38] Harris W., Ready D., Lipson E., Hudspeth A., Stark W. (1977). Vitamin A deprivation and *Drosophila* photopigments. Nature.

[39] Wang T., Jiao Y., Montell C. (2007). Dissection of the pathway required for generation of vitamin A and for *Drosophila* phototransduction. J. Cell Biol..

[40] Lane M.A., Bailey S.J. (2005). Role of retinoid signalling in the adult brain. Prog. Neurobiol..

[41] Travis G.H., Golczak M., Moise A.R., Palczewski K. (2007). Diseases caused by defects in the visual cycle: Retinoids as potential therapeutic agents. Annu. Rev. Pharmacol. Toxicol..

[42] Baik L.S., Recinos Y., Chevez J.A., Au D.D., Holmes T.C. (2019). Multiple phototransduction inputs integrate to mediate UV light-evoked avoidance/attraction behavior in *Drosophila*. J. Biol. Rhythm..

[43] Wang D., Yang G., Chen W. (2021). Diel and circadian patterns of locomotor activity in the adults of diamondback moth (*Plutella xylostella*). Insects.

[44] Mazzotta G., Rossi A., Leonardi E., Mason M., Bertolucci C., Caccin L., Spolaore B., Martin A.J.M., Schlichting M., Grebler R. (2013). Fly cryptochrome and the visual system. Proc. Natl. Acad. Sci. USA.

