# Supplementary material for: An LW-Opsin Mutation Changes the Gene Expression of the Phototransduction Pathway: A Cryptochrome1 Mutation Enhances the Phototaxis of Male Plutella xylostella (Lepidoptera: Plutellidae)
Source: Insects. 2023 Jan 12;14(1):72. doi: 10.3390/insects14010072 (PMC9860677; doi:10.3390/insects14010072)
Supplement: Supplementary file 1 [file insects-14-00072-s001.zip › insects-2081471-supplementary.pdf]

Supplementary Information

Table S1. RT-qPCR primers

Gene ID	Gene name	Forward primer (5'-3')	Reverse primer (5'-3')
Px003840	*actin*	ACGAGGCCCAGAGCAAGAGAG	TGGTGCCAGATCTTCTCCATG
Px005510	*inaD*	CCTCGGCATCATGATCATCGA	CTGCCAGTATCATGTCACCTA
Px005830	*ninaA*	ATGGTTCAAGCTGGTGATGTG	AATCCTGCCAGAGTGTGTTTG
Px006397	*cry1*	GACAGCCAGTTCAAGAAGCACG	CGAAGCAGAGCTTGCGGAT
Px009837	*LW-opsin*	ATCTGGCTGTTCTCACTCGC	CTTGCTCAGGTAGTCGGTGC
Px009888	*trpl*	AAGCGCACCGTAAGAAGCACA	TCCTTGGTCTGCACCTCCATG
Px009889	*trp*	ATTTGGTACGAAGGACTGCCC	GGCAGATGAACTTGACGAAGG
Px014948	*arr1*	AACAACAGCAGCAAGGTGGT	CATCCGTCCTGCGTCTCGA
Px018010	*CaM*	AGACGGCAACGGCACGATAGA	AGATGAAGCCGTTGCCGTCCT

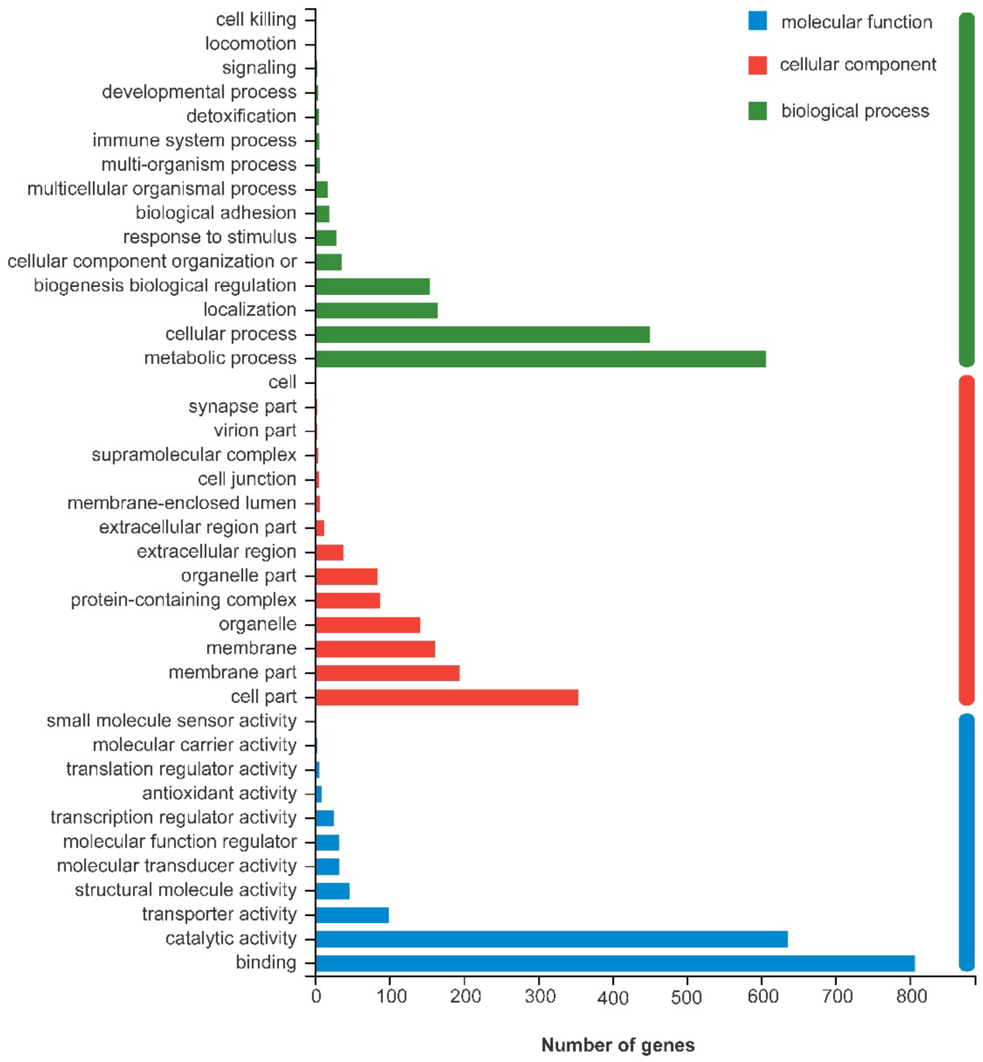

Figure S1. GO cluster diagram of DEGs

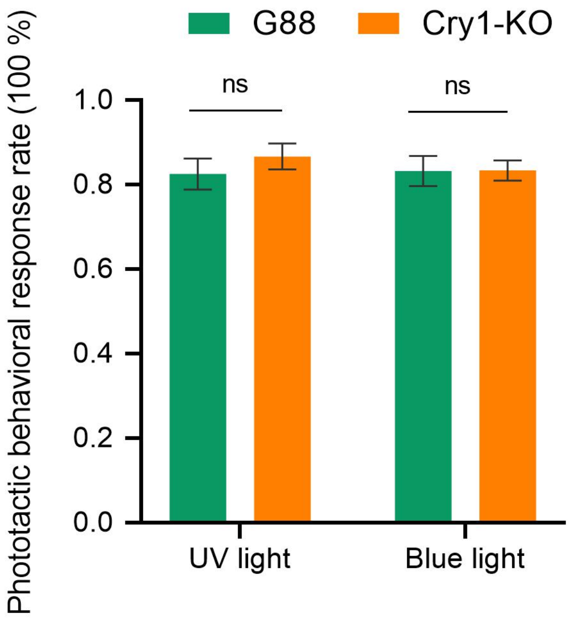

Figure S2. The influence of *cry1* mutation on the phototaxis of *P. xylostella* female
